# Comparative Angiogenic Potential of Collagen and Polytetrafluoroethylene Guided Tissue Regeneration Membranes: An Experimental Chick Embryo Chorioallantoic Membrane Assay Study

**DOI:** 10.7759/cureus.111143

**Published:** 2026-06-19

**Authors:** Roopal Gupta, Vidya Dodwad, Pooja Pharne, Nishita Bhosale, Devashri Newaskar, Sanpreet S Sachdev

**Affiliations:** 1 Periodontology, Bharati Vidyapeeth (Deemed to be University) Dental College and Hospital, Pune, IND; 2 Oral Pathology and Microbiology, Bharati Vidyapeeth (Deemed to be University) Dental College and Hospital, Navi Mumbai, IND

**Keywords:** angiogenesis, collagen membrane, gingival stem cells, guided tissue regeneration, ptfe membrane

## Abstract

Background

Angiogenesis is an important biological event in periodontal regeneration, as early vascular support is necessary for oxygen delivery, nutrient transport, and tissue healing. Guided tissue regeneration (GTR) membranes may influence this vascular response depending on their material properties.

Aim

To compare the angiogenic response of resorbable collagen and non-resorbable polytetrafluoroethylene (PTFE) GTR membranes using the chick embryo chorioallantoic membrane (CAM) assay.

Materials and methods

Thirty fertilized chicken eggs were divided equally into three groups: control, collagen membrane, and PTFE membrane. On Day 3 of incubation, the respective membranes were placed onto the CAM surface, while no membrane was placed in the control group. On Day 7, macroscopic images were obtained and analyzed using Wimasis Image Analysis software (Wimasis, Onimagin Technologies SCA, Córdoba, Spain). Vessel density, total vessel network length, and branching points were compared using Welch analysis of variance and Games-Howell post hoc test.

Results

PTFE showed significantly higher vessel density, total vessel network length, and branching points than both the control and collagen groups (p < 0.001). The collagen group showed significantly higher branching points than the control group, but vessel density and total vessel network length were not significantly different between these two groups.

Conclusion

PTFE demonstrated a stronger angiogenic response than collagen in the CAM model. These findings suggest material-dependent differences in early vascular response, but further histological, animal, and clinical studies are required before clinical conclusions can be made.

## Introduction

Periodontitis is a chronic inflammatory disease that causes progressive destruction of the periodontal ligament, cementum, and alveolar bone, ultimately compromising tooth support and function if not adequately managed [1]. Although conventional periodontal therapy can arrest disease progression, the ideal goal of treatment is periodontal regeneration, defined by the formation of new cementum, functionally oriented periodontal ligament fibers, and new alveolar bone rather than repair by a long junctional epithelium.

Guided tissue regeneration (GTR) was introduced to promote selective cell repopulation during periodontal wound healing. In this approach, a barrier membrane is placed between the gingival flap and root surface to exclude rapidly migrating epithelial and gingival connective tissue cells, thereby allowing periodontal ligament and bone-derived cells to participate in regeneration [2,3]. Clinical evidence supports the use of GTR in periodontal intrabony defects; however, treatment outcomes are influenced by several membrane-related properties, including biocompatibility, space maintenance, tissue integration, degradation behavior, and biological interaction with the healing tissues [4].

GTR membranes are broadly categorized as resorbable and non-resorbable. Collagen-based resorbable membranes are commonly used because of their favorable handling characteristics, biocompatibility, hemostatic potential, and avoidance of a second surgical procedure for membrane removal. In contrast, non-resorbable polytetrafluoroethylene (PTFE) membranes provide superior structural stability and space-maintaining capacity, but they require retrieval and may be associated with exposure-related complications. Previous experimental studies have shown that membrane composition and surface characteristics can influence cellular attachment, proliferation, and differentiation, suggesting that GTR membranes may have biological effects beyond their passive barrier function [5,6]. In addition to supporting cell exclusion and space maintenance, the membrane surface may influence early vascular events by affecting blood clot stability, tissue contact, endothelial cell migration, and local vascular sprouting. These membrane-induced vascular responses are clinically relevant because angiogenesis contributes to early wound organization and may influence the quality of subsequent periodontal regeneration.

Angiogenesis is a critical event in periodontal and bone regeneration because newly formed tissues require an early vascular supply for oxygen delivery, nutrient transport, inflammatory-cell regulation, progenitor-cell recruitment, and removal of metabolic waste [2,4]. The initial vascular response around a regenerative membrane is influenced not only by the healing tissue but also by membrane-related factors such as material composition, surface topography, porosity, degradation behavior, and structural stability. A membrane that permits or promotes favorable vascular organization may support the early healing environment, whereas inadequate vascularization may compromise tissue maturation and reduce regenerative potential [4]. Therefore, comparative evaluation of the angiogenic behavior of different GTR membranes can provide useful preclinical information regarding their biological performance.

The chick embryo chorioallantoic membrane (CAM) assay is a well-established experimental model for evaluating angiogenesis and biomaterial biocompatibility. It allows direct visualization of vascular responses, quantitative assessment of vessel formation, and rapid screening of test materials in a living vascular environment before more complex animal or clinical studies are undertaken [7]. Although collagen and PTFE membranes are widely used in periodontal regenerative procedures, direct comparative evidence on their membrane-induced angiogenic response remains limited, particularly using standardized image-based CAM assay evaluation. Therefore, the present study aimed to compare the angiogenic response induced by resorbable collagen and non-resorbable PTFE GTR membranes using the CAM assay. The specific objectives were to evaluate and compare vessel density, total vessel network length, and vascular branching points among control, collagen membrane, and PTFE membrane groups.

## Materials and methods

Study design

This in ovo experimental study was conducted to compare the angiogenic response produced by resorbable collagen and non-resorbable PTFE GTR membranes using the CAM assay. The study used fertilized chicken eggs for pre-hatching CAM-based angiogenesis assessment. No human participants, human tissue samples, mammalian animals, or clinical interventions were involved in the experimental work. The study included three independent groups: a control group, a collagen membrane group, and a PTFE membrane group. The control group consisted of CAMs exposed under identical experimental conditions without the placement of any membrane material. Thirty fertilized chicken eggs were used, with 10 eggs allocated to each group. Each egg was considered an independent experimental unit for image analysis and statistical comparison. The study protocol was approved by the Institutional Ethics Committee of Bharati Vidyapeeth (Deemed to be University) Dental College and Hospital, Pune, India, with reference letter number BVDU/IEC/R4/11/23-24, dated 15/05/2024.

Materials

The test materials consisted of a resorbable collagen GTR membrane, Periocol® (Eucare Pharmaceuticals Pvt. Ltd., Chennai, India), and a non-resorbable PTFE barrier membrane, OD Biomem® PTFE membrane (B&MEDI Co., Ltd., Seoul, South Korea). Periocol® is a type I collagen membrane derived from bovine xenogenic collagen, while OD Biomem® is a non-resorbable PTFE membrane used for guided tissue and bone regeneration. Both membranes were used from their commercially supplied sterile packaging and trimmed into standardized 2×2 mm specimens under aseptic conditions before placement on the CAM surface. The experimental procedure was carried out using fertilized chicken eggs, an egg incubator, sterile marking instruments, Castroviejo scissors, sterile forceps, a syringe for albumin aspiration, sterile adhesive tape, and a digital imaging system for macroscopic photography. Quantitative vascular assessment was performed using Wimasis Image Analysis software, which provided image-wise values for vessel density, total vessel network length, and branching points.

Experimental grouping

The fertilized chicken eggs were divided equally into three groups. In the control group, the CAM was exposed under the same experimental conditions, but no membrane material was placed. In the collagen group, a standardized 2×2 mm specimen of Periocol® collagen membrane was placed onto the CAM surface. In the PTFE group, a standardized 2×2 mm specimen of OD Biomem® PTFE membrane was placed onto the CAM surface. The same incubation, windowing, handling, sealing, and imaging protocol was followed for all groups to maintain procedural consistency.

CAM assay procedure

On Day 0, the fertilized eggs were examined by candling to identify viable embryos and locate the air sac. The air sac was marked externally, and the eggs were incubated horizontally at 37.5°C with approximately 65% relative humidity. The eggs were handled carefully during incubation to avoid injury to the developing embryo and extraembryonic membranes.

**Figure 1:**
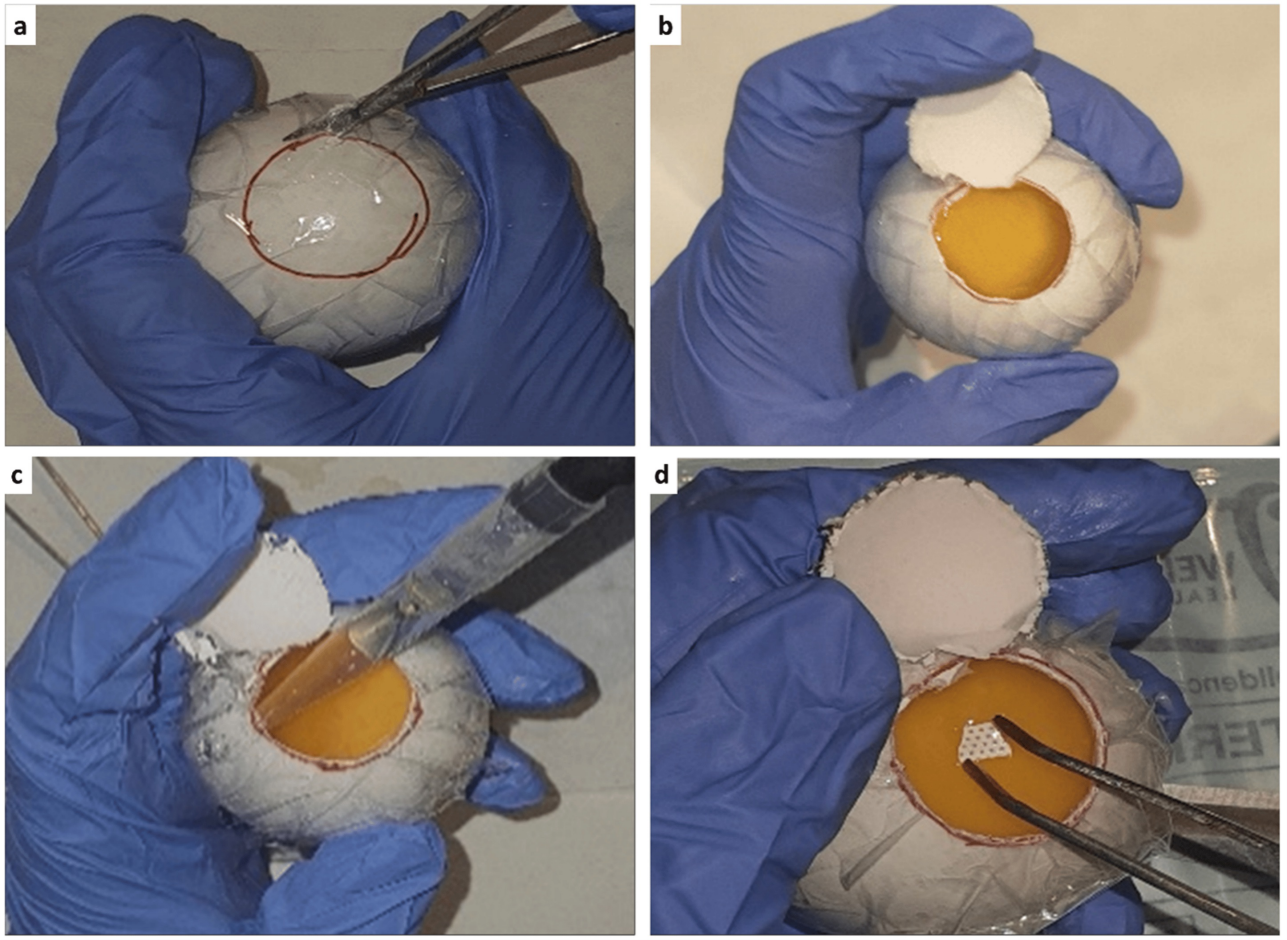
Steps of the chorioallantoic membrane (CAM) assay procedure a) Candling of the fertilized chicken egg; b) creation of a small eggshell window; c) aspiration of 3 mL albumin; d) placement of the guided tissue regeneration membrane onto the CAM

On Day 3 of incubation, the eggshell surface over the marked air sac was disinfected with 70% ethanol under aseptic conditions. A small window was created using Castroviejo scissors. Approximately 3 mL of albumin was aspirated carefully to lower the developing CAM and create adequate working space for membrane placement. In the collagen and PTFE groups, standardized 2 × 2 mm membrane specimens were gently placed onto the CAM surface using sterile forceps (Figure 1). In the control group, the CAM was exposed in the same manner, but no membrane material was placed. Following membrane placement, the window was sealed with sterile adhesive tape, and the eggs were returned to the incubator under the same temperature and humidity conditions.

On Day 7 of incubation, the sealed windows were reopened, and the CAMs were exposed for imaging. Macroscopic photographs were obtained from each egg under standardized imaging conditions. The area surrounding the test membrane, or the corresponding area in the control group, was selected for quantitative angiogenesis assessment. Images were obtained under similar lighting, distance, and field-selection conditions for all specimens to maintain consistency during software-based vascular analysis.

Image analysis and outcome assessment

**Figure 2:**
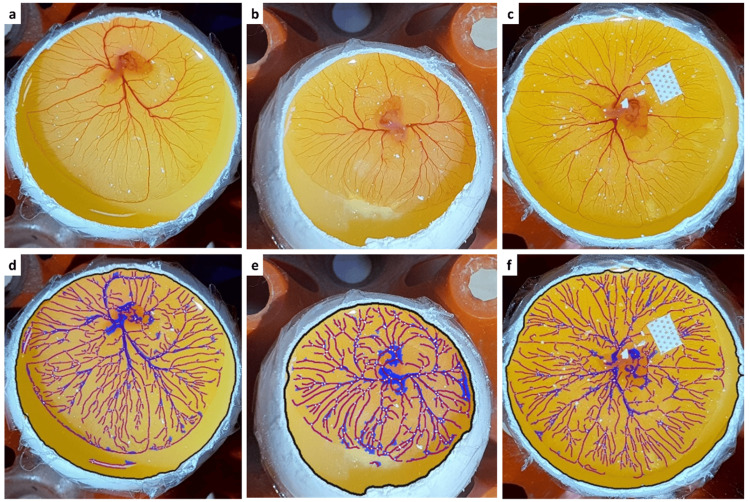
Representative chorioallantoic membrane images and Wimasis image analysis. a) Control group; b) collagen membrane group; c) polytetrafluoroethylene membrane group; d–f) corresponding Wimasis vessel analysis overlays for the respective groups. Small dots indicate branching points, and lines indicate tracked blood vessels.

The CAM images were analyzed using Wimasis Image Analysis software (Wimasis, Onimagin Technologies SCA, Córdoba, Spain). The primary outcome variables were vessel density, total vessel network length, and branching points. Vessel density was expressed as the percentage of the analyzed area occupied by blood vessels. Total vessel network length was recorded in pixels and represented the cumulative length of the vascular network detected within the selected field. Branching points represented the number of vascular branch points identified within the same region of interest (Figure 2). The same image-analysis method was applied across all groups, and the final analysis was based on the individual egg-wise values generated from the Wimasis output. To reduce measurement bias, image files were coded before analysis, and vascular parameters were recorded from the software output before group-wise statistical comparison.

Statistical analysis

Data were entered and analyzed using IBM SPSS Statistics version 25.0 (IBM Corp., Armonk, NY, USA). Each egg was considered an independent experimental unit, and statistical analysis was performed using egg-wise image-analysis values. Continuous variables were expressed as mean and standard deviation. The Shapiro-Wilk test was used to assess normality, and Levene’s test was used to assess homogeneity of variance. Since the study compared three independent groups and the assumption of equal variance was not consistent across angiogenic parameters, Welch analysis of variance was used for intergroup comparison. Overall test values were reported as Welch F values. Pairwise intergroup comparisons were performed using the Games-Howell post hoc test, and pairwise test values were reported as Games-Howell q values with corresponding p-values. A p-value of less than 0.05 was considered statistically significant. 

## Results

The final analysis included 30 CAM images, with 10 images each in the control, collagen membrane, and PTFE membrane groups. Angiogenesis was assessed using vessel density, total vessel network length, and branching points. Since the assumption of equal variance was not consistent across the angiogenic parameters, Welch analysis of variance was used for overall intergroup comparison, followed by Games-Howell post hoc testing.

The mean vessel density was highest in the PTFE membrane group, followed by the collagen membrane group and the control group. The overall difference among the three groups was statistically significant (p < 0.001). Total vessel network length also differed significantly among the groups (p < 0.001), with the highest mean value in the PTFE membrane group. Branching points showed a statistically significant intergroup difference as well (p < 0.001), with the PTFE membrane group showing the highest mean number of branching points. The detailed group-wise values are presented in Table 1.

**Table 1:** Comparison of angiogenic parameters among the study groups Values are expressed as mean ± standard deviation. PTFE: polytetrafluoroethylene

Parameter	Control group, n = 10	Collagen membrane group, n = 10	PTFE membrane group, n = 10	Welch F test value	p-value
Vessel density (%)	22.39 ± 2.51	23.43 ± 2.53	32.77 ± 3.27	34.20	<0.001
Total vessel network length (pixels)	1854.34 ± 395.08	2044.72 ± 540.89	5200.25 ± 781.35	73.02	<0.001
Branching points (count)	209.60 ± 72.08	437.90 ± 71.42	566.00 ± 34.67	98.21	<0.001

Games-Howell post hoc analysis showed that vessel density was significantly higher in the PTFE membrane group than in the control group (mean difference = 10.37, p < 0.001) and the collagen membrane group (mean difference = 9.33, p < 0.001). The difference in vessel density between the collagen membrane and control groups was not statistically significant (mean difference = 1.04, p = 0.633). For total vessel network length, the PTFE membrane group showed significantly higher values than the control group (mean difference = 3345.90 pixels, p < 0.001) and collagen membrane group (mean difference = 3155.53 pixels, p < 0.001), while the difference between the collagen membrane and control groups was not significant (mean difference = 190.37 pixels, p = 0.649). For branching points, the collagen membrane group showed significantly higher values than the control group (mean difference = 228.30, p < 0.001), and the PTFE membrane group showed significantly higher values than both the control group (mean difference = 356.40, p < 0.001) and the collagen membrane group (mean difference = 128.10, p < 0.001). The complete pairwise comparisons are shown in Table 2.

**Table 2:** Pairwise Games-Howell post hoc comparison of angiogenic parameters Mean difference is calculated as the first-mentioned group minus the comparator group. Games-Howell post hoc test was applied after Welch analysis of variance. PTFE: polytetrafluoroethylene

Parameter	Pairwise comparison	Mean difference	Games-Howell q value	p-value
Vessel density (%)	Collagen membrane vs Control	1.04	1.30	0.633
Vessel density (%)	PTFE membrane vs Control	10.37	11.26	<0.001
Vessel density (%)	PTFE membrane vs Collagen membrane	9.33	10.10	<0.001
Total vessel network length (pixels)	Collagen membrane vs Control	190.37	1.27	0.649
Total vessel network length (pixels)	PTFE membrane vs Control	3345.90	17.09	<0.001
Total vessel network length (pixels)	PTFE membrane vs Collagen membrane	3155.53	14.85	<0.001
Branching points (count)	Collagen membrane vs Control	228.30	10.06	<0.001
Branching points (count)	PTFE membrane vs Control	356.40	19.93	<0.001
Branching points (count)	PTFE membrane vs Collagen membrane	128.10	7.22	<0.001

## Discussion

The present study evaluated the angiogenic response of resorbable collagen and non-resorbable PTFE GTR membranes using the CAM assay. The PTFE membrane showed significantly higher vessel density, total vessel network length, and branching points than both the collagen membrane and the control groups. The collagen membrane showed an intermediate response, with a significant increase in branching points compared with the control group, although vessel density and total vessel network length did not differ significantly between these two groups. These findings indicate that the two membrane materials produced different early vascular responses in the CAM model.

Angiogenesis is an important component of periodontal wound healing because newly forming tissues require vascular support for oxygen delivery, nutrient diffusion, cellular recruitment, and removal of metabolic waste. Periodontal regeneration is therefore influenced not only by epithelial exclusion and space maintenance, but also by the quality of the local healing environment, including vascular stability and tissue organization [8]. In oral wound healing, improved oxygenation and vascularization are closely associated with more favorable tissue repair, particularly during the proliferative and remodeling phases [9]. In this context, the higher angiogenic response observed around PTFE may reflect a stronger local vascular reaction to the membrane surface under the present experimental conditions.

The CAM assay was selected because it provides a highly vascular living membrane in which biomaterial-associated vascular changes can be directly visualized and quantified. It has been widely used for evaluating angiogenic and anti-angiogenic responses, as well as for testing biomaterials in a biologically active vascular environment [10-12]. However, CAM-based findings require careful interpretation because vascular measurements may be influenced by embryo age, region of interest, imaging conditions, and image-analysis method [13]. In the present study, these sources of variation were reduced by using the same incubation period, membrane exposure duration, imaging protocol, and software-based vascular analysis across all groups. The use of Wimasis image analysis also allowed objective quantification of vessel density, total vessel network length, and branching points, which are commonly used angiogenic parameters in biomaterial-related CAM studies [14].

The stronger angiogenic response observed with PTFE may be related to its non-resorbable nature, structural stability, and ability to maintain a defined surface interface during the observation period. Membrane performance in guided tissue and bone regeneration depends on several material-related factors, including composition, porosity, surface characteristics, mechanical stability, degradation behavior, and tissue interaction [15]. Recent reviews have emphasized that barrier membranes should not be considered only passive occlusive materials, as membrane design can influence biological events at the healing site, including vascular response, tissue integration, and host-material interaction [16-18]. Therefore, the greater vascular response around PTFE in this study may represent a material-specific host response rather than a purely mechanical effect.

Although PTFE is generally considered chemically stable and relatively inert, experimental evidence suggests that it can still produce a measurable tissue response. Korzinskas et al. reported that dense PTFE membranes showed acceptable biocompatibility but were associated with detectable macrophage and tissue responses around the material [19]. More recent work on occlusive membranes for periodontal regeneration has also shown that membrane surfaces can influence local healing events in inflamed tissue defects, including tissue integration and inflammatory behavior [20]. The present findings are consistent with this broader concept that non-resorbable PTFE membranes may participate in early biological events around the regenerative site, even though their principal clinical advantage remains space maintenance and barrier function.

The collagen membrane findings should also be interpreted carefully. Collagen membranes are widely used because of their biocompatibility, resorbable nature, hemostatic potential, and ease of clinical handling [21]. However, collagen membranes vary considerably depending on their source, cross-linking method, degradation profile, and surface architecture. Schwarz et al. showed that native and cross-linked collagen membranes can demonstrate different angiogenesis patterns after implantation [22], while Rothamel et al. reported that cross-linking can alter collagen membrane degradation and tissue response [23]. Similarly, Sehgal et al. showed that different collagen barrier membranes may vary in their occlusive and proliferative properties [24]. In the present study, the collagen membrane increased vascular branching but did not significantly increase vessel density or total vessel length compared with the control group, suggesting a limited but measurable effect on vascular organization during the observation period.

The clinical relevance of these results should not be overstated. A stronger angiogenic response in the CAM assay does not prove that PTFE will produce superior periodontal regeneration clinically. Clinical outcomes after GTR are influenced by several factors that are not reproduced in the CAM model, including periodontal defect morphology, flap closure, bacterial contamination, wound stability, membrane exposure, patient-related factors, and the need for membrane retrieval [25]. In addition, this study did not assess membrane thickness, porosity, surface roughness, degradation profile, histological vascular ingrowth, or molecular angiogenic markers. A formal a priori sample size calculation was not performed, and the sample size of 10 eggs per group was based on experimental feasibility. Therefore, the findings should be considered preclinical evidence of differential angiogenic behavior rather than direct evidence of clinical superiority. Future studies should include detailed membrane characterization, histological and immunohistochemical validation of angiogenesis, molecular markers such as vascular endothelial growth factor or CD31, larger sample sizes with power calculation, and periodontal defect models before drawing firm clinical conclusions regarding regenerative performance.

## Conclusions

Within the limitations of this CAM assay-based study, the PTFE GTR membrane demonstrated a stronger angiogenic response than the collagen membrane, as reflected by significantly higher vessel density, total vessel network length, and branching points. The collagen membrane showed a limited angiogenic effect, with a significant increase in vascular branching but no significant improvement in vessel density or total vessel network length compared with the control. Further studies with histological validation, membrane characterization, and periodontal defect models are required to determine whether these early angiogenic differences have clinical relevance for periodontal regeneration.
